# Prediabetes Induces More Severe Acute COVID-19 Associated With IL-6 Production Without Worsening Long-Term Symptoms

**DOI:** 10.3389/fendo.2022.896378

**Published:** 2022-07-08

**Authors:** Icaro Bonyek-Silva, Thiago Cerqueira-Silva, Sara Nunes, Antônio Fernando Araújo Machado, Márcio Rivison Silva Cruz, Blenda Pereira, Leilane Estrela, Jéssica Silva, Ananda Isis, Aldina Barral, Pablo Rafael Silveira Oliveira, Ricardo Khouri, C. Henrique Serezani, Cláudia Brodskyn, Juliana Ribeiro Caldas, Manoel Barral-Netto, Viviane Boaventura, Natalia Machado Tavares

**Affiliations:** ^1^ Gonçalo Moniz Institute (IGM), Oswaldo Cruz Foundation (FIOCRUZ), Salvador, Brazil; ^2^ Medical School, Federal University of Bahia (FAMEB-UFBA), Salvador, Brazil; ^3^ Federal Institute of Education, Science and Technology Baiano, Xique-Xique, Brazil; ^4^ Faculty of Santa Cruz of Bahia (FSC), Nursing School, Itaberaba, Brazil; ^5^ School of Health Sciences, Salvador University (UNIFACS), Salvador, Brazil; ^6^ National Institute of Science and Technology (INCT), Institute of Investigation in Immunology (iii-INCT), São Paulo, Brazil; ^7^ Institute of Biological Sciences, Federal University of Bahia (IBio-UFBA), Salvador, Brazil; ^8^ Division of Infectious Diseases, Department of Medicine, Vanderbilt University Medical Center, Nashville, TN, United States; ^9^ Critical Care Unit, São Rafael Hospital – Rede d’Or, Salvador, Brazil; ^10^ Bahiana School of Medicine and Public Health – EBMSP, Campus Brotas, Salvador, Brazil

**Keywords:** prediabetes, inflammation, COVID-19, long COVID, IL-6

## Abstract

**Aims:**

Pre-existing conditions, such as age, hypertension, obesity, and diabetes, constitute known risk factors for severe COVID-19. However, the impact of prediabetes mellitus (PDM) on COVID-19 severity is less clear. This study aimed to evaluate the influence of PDM in the acute and long-term phases of COVID-19.

**Materials and methods:**

We compared inflammatory mediators, laboratory and clinical parameters and symptoms in COVID-19 patients with prediabetes (PDM) and without diabetes (NDM) during the acute phase of infection and at three months post-hospitalization.

**Results:**

Patients with PDM had longer hospital stays and required intensive care unit admission more frequently than NDM. Upon hospitalization, PDM patients exhibited higher serum levels of interleukin 6 (IL-6), which is related to reduced partial pressure of oxygen (PaO_2_) in arterial blood, oxygen saturation (SpO_2_) and increased COVID-19 severity. However, at three months after discharge, those with PDM did not exhibit significant alterations in laboratory parameters or residual symptoms; however, PDM was observed to influence the profile of reported symptoms.

**Conclusions:**

PDM seems to be associated with increased risk of severe COVID-19, as well as higher serum levels of IL-6, which may constitute a potential biomarker of severe COVID-19 risk in affected patients. Furthermore, while PDM correlated with more severe acute-phase COVID-19, no long-term worsening of sequelae was observed.

## Introduction

The deadly Coronavirus disease 2019 (COVID-19) pandemic due to the novel Severe Acute Respiratory Syndrome Coronavirus 2 (SARS-CoV-2) continues to present an enormous challenge to health systems worldwide. Gaps in our understanding of COVID-19 have undoubtedly exacerbated the death toll of over 6 million people worldwide, according to the World Health Organization.

The impact of the COVID-19 pandemic has been largely accentuated by the transmission capabilities of SARS-CoV-2. This new coronavirus interacts with different host cells by binding its viral SPIKE protein to the host’s ACE2 receptor, mediated by proteases such as transmembrane serine protease 2 (TMPRSS2) and FURIN (1).

The spectrum of COVID-19 presentation varies widely, from mild to moderate and severe clinical forms. The severe form of disease occurs predominantly in elderly, hypertensive, obese, and diabetic individuals (2). In the context of diabetes, researchers around the world have been struggling to identify the mechanism underlying increased severe COVID-19 risk in these individuals. Recently, our group demonstrated the involvement of the Leukotriene B4 (LTB_4_) pathway in severe cases of COVID-19 in individuals with diabetes, and reported increased expression of ACE2 and TMPRSS2 in peripheral blood mononuclear cells (3). Other studies have also highlighted the importance of increased expression of these SARS-CoV-2 gateway receptors to the pathogenesis of COVID-19 using different cell types placed under hyperglycemic conditions (4–7).

Based on preliminary exploratory study, prediabetes also appears to be a risk factor for severe COVID-19 (8–10). However, the mechanisms that lead to disease exacerbation remain unknown. Moreover, the potential for these patients to develop residual symptoms after acute phase of the COVID-19 has yet to be investigated. Thus, we sought to assess the involvement of inflammatory mediators in PDM individuals with severe COVID-19 requiring hospitalization. Our results indicate that COVID-19 patients with PDM experience a greater degree of lung injury, require prolonged hospitalization and intensive care admission. However, PDM does not seem to impact the long-term prevalence of symptoms post-acute COVID-19. Finally, our results suggest that serum levels of IL-6 may represent a promising marker of unfavorable outcomes associated with PDM in COVID-19 patients.

## Materials and Methods

### Study Approval

This study followed the principles of the Declaration of Helsinki. The Institutional Board for Ethics in Human Research at the Gonçalo Moniz Institute, Oswaldo Cruz Foundation (CAAE 36199820.6.0000.0040), and Irmã Dulce Social Works (CAAE 33366020.5.0000.0047) approved this study. Participants gave informed consent previous to any data and sample collection.

### Patients

Patients diagnosed positive for COVID-19, based on the positivity of molecular test (RT-qPCR) or clinical history for COVID-19. In the study of the acute phase of COVID-19, patients were diagnosed through RT-qPCR or clinical plus radiologic criteria and were admitted to Ernesto Simões Filho General and Memorial Hospital, Salvador, Brazil, from July 2020 to February 2021. Forty-two patients were enrolled in this study, 23 without diabetes (NDM) and 19 with prediabetes (PDM). To analyze the consequences of COVID-19, patients with confirmed SARS-CoV-2 infection through RT-PCR/Lateral-flow or serologic tests, three hundred and three patients were enrolled 3 months after symptom onset (acute phase between August 2020 and May 2021) from Octávio Mangabeira Specialized Hospital, Salvador, Brazil, of which 130 are without diabetes and 173 with prediabetes. According to the Brazilian Diabetes Society guidelines, 2019-2020 (Lyra et al., 2020), in this study, patients with HbA1c between ≥ 39 mmol/mol (5.7%) and < 48 mmol/mol (6.5%) were considered with prediabetes (PDM) and patients with values < 5.7% were considered without diabetes (NDM). The score to assess mobility impairment in the post COVID-19 phase was based on EuroQol questionnaire. Clinical data from all patients were obtained on admission from medical records and managed on the REDCap platform. Patients who did not agree to sign the free and informed consent, were pregnant, who did not have the value of glycated hemoglobin, had symptoms for >14 days, and had been in the hospital for >48 h were excluded of this study.

### Quantification of Inflammatory Mediators

Blood samples from all patients were collected at admission. Plasma was separated to quantify inflammatory mediators. Based on the highlight of specific inflammatory mediators in the outcome of COVID-19 (Pérez et al., 2021; Tay et al., 2020), serum levels of Tumor Necrosis Factor alpha (TNF-α), Interleukin 6 (IL-6) and LTB_4_ (Cayman Chemical, USA) were evaluated using Enzyme Linked Immunosorbent Assay (ELISA).

### Gene Expression Analysis

Total RNA was extracted from peripheral blood mononuclear cells (PBMCs) collected at admission using miRNeasy Mini Kit (QIAGEN, Hilden, Germany) according to the manufacturer’s guidelines. Relative expression of ACE2 (assay ID Hs.PT.58.27645939); transmembrane serine protease 2 (TMPRSS2) (assay ID Hs.PT.58.4661363); furin, paired basicamino acid cleaving enzyme (FURIN) (assay ID Hs.PT.58.1294962 were analyzed. cDNA synthesis was performed using the SuperScript III Reverse Transcriptase Kit (Invitro-gen, Carlsbad, CA). Then, cDNA was amplified by quantitative real-time PCR using the SYBR Green PCR Master Mix (Thermo Fisher Scientific, Waltham, MA). Relative gene expression is shown as the fold change between the NDM and PDM groups using b-actin as housekeeping gene (ACTB) (assay ID Hs.PT.39a.22214847). All primers were pur-chased from Integrated DNA Technologies (Coralville, IA).

### Statistical Analysis

Data are presented as mean and SD or median and interquartile range values for numerical variables and proportions (%) for categorical variables. For variables with normal distribution, we used Student’s t-test (two groups). For non-normal distribution, we used Mann–Whitney test (two groups), Kruskal–Wallis with Dunn’s post-test (three or more groups), and the Spearman test we used for correlations analysis. Chi-Square or Fisher’s exact test was used to compare proportions. The hierarchical clustering analysis was performed based on the average of the Euclidean distance between symptoms and patients splitted by group using Orange software version 3.28 with patients without missing data. Outliers were identified using ROUT method (Q=1%). All tests were conducted using Prism 8 software (GraphPad, USA). Differences were considered statistically significant when *p* < 0.05, or adj. *p* < 0.05 for multiple comparisons.

## Results

### High Serum Levels of IL-6 and Severe COVID-19 Outcomes in Prediabetic Patients

This study evaluated 42 patients with COVID-19 in the acute phase of infection: 23 (10 F: 13 M; median age 54 years) non-diabetic (NDM) controls, and 19 prediabetic individuals (06 F: 13 M; median age 67 years). The groups were proportionately similar with regard to comorbidities and symptoms (**Table 1**) and no differences were seen in drug therapy during hospitalization between the groups (see **Supplementary Table 1**).

**Table 1:** Clinical characteristics among non-diabetic (NDM) and prediabetic (PDM) patients with Coronavirus Disease 2019 (COVID-19) stratified according to low or high IL-6 production.

	All patients, *n*			PDM patients, *n*		
CHARACTERISTICS	NDM (n = 23)	PDM (n = 19)	*p value*	LOW (n = 7)	HIGH (n = 12)	*p value*
Male, n/N (%)	13/23 (56%)	13/18 (72%)	0.300	4/7 (57%)	9/11 (82%)	0.326
Age, mean ± SD	54 ± 19	67 ± 16	0.053	65 ± 17	67 ± 16	0.336
Hb1Ac, median (IQR) mmol/mol%	34 (32 - 38)5.3 (5.1 - 5.6)	42 (39 - 45)6.0 (5.7 - 6.3)	<0.0001	41 (39 - 44)5.9 (5.7 - 6.2)	42 (39 - 45)6.0 (5.7 - 6.3)	0.334
Onset of symptoms prior to hospitalization (in days), median (IQR), N	7 (5-13)(N=11/23)	4 (2-6)(N=12/19)	0.086	2 (2-4)(N=5/7)	6 (3-13)(N=7/12)	0.093
**Comorbidities n/N (%)**						
Obesity	0/13 (0%)	4/14 (28%)	0.097	2/5 (40%)	2/9 (22%)	0.580
Hypertension	10/18 (55%)	6/15 (40%)	0.373	3/5 (60%)	3/9 (33%)	0.580
COPD	1/11 (9%)	2/12 (17%)	>0.999	0/3 (0%)	2/9 (22%)	>0.999
**Symptoms n/N (%)**						
Fever	11/17 (65%)	6/12 (50%)	0.428	2/5 (40%)	4/7 (57%)	>0.999
Cough	14/20 (70%)	12/14 (86%)	0.422	6/6 (100%)	6/8 (75%)	0.472
Dyspnea	12/19 (63%)	15/16 (94%)	0.090	6/6 (100%)	9/10 (90%)	>0.999
**Outcome n/N (%)**						
Death	3/22 (14%)	6/17 (35%)	0.456	1/6 (16%)	5/11 (45%)	0.333

COPD, Chronic Obstructive Pulmonary Disease; n, Total number of patients; N, Number of patients with information available.

**Figure 1:**
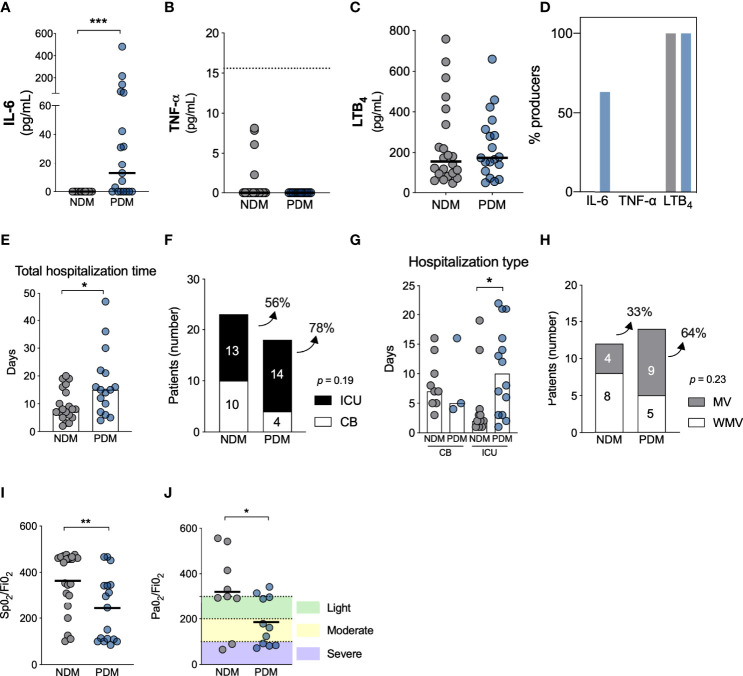
COVID-19 disease is more severe in individuals with prediabetes. Systemic levels of IL-6 **(A)**, TNF-a **(B)** and LTB_4_ **(C)** in individuals without diabetes (NDM) and prediabetes (PDM) with COVID-19. **(D)** Percentage of individuals producing inflammatory mediators in NDM and PDM with COVID-19. **(E)** Total hospitalization time between NDM and PDM individuals. **(F)** Number and percentage of NDM and PDM individuals admitted to clinical beds (CB) (white) or intensive care unit (ICU) (black) due to COVID-19. **(G)** Days of hospitalization in ICU or CB in NDM and PDM with COVID-19. **(H)** Number and percentage of patients who required (black) mechanical ventilation or not (white) between NDM and PDM group. **(I)** SpO_2_/FiO_2_ and **(J)** PaO_2_/FiO_2_ ratio in NDM and PDM patients with COVID-19. [**(A, B, E)** = Mann Whitney test]; [**(F, H)** = Fisher’s exact test]; [**(G)** = Kruskal-Wallis with Dunn’s post-test); [**(I, J)** = Unpaired t-test]. *p < 0.05; **p < 0.01; ***p < 0.001.

Our analysis of inflammatory mediators revealed that prediabetic patients demonstrated increased IL-6 levels (*p* = 0.0001) during acute COVID-19; however, no differences were seen in TNF-a or LTB_4_ (**Figures 1A-C**). **Figure 1D** shows the percentage of patients who were producers of detectable levels of inflammatory mediators. While both groups produced LTB_4_ and none produced TNF-a, approximately 63% of PDM exhibited high levels of IL-6 compared to NDM.

We observed that PDM patients with COVID-19 required extended hospital stays [15 days (IQR 8-22)] compared to NDM [8 days (IQR 5-15)], as illustrated in **Figure 1E** (*p* = 0.044). Among the PDM COVID-19 patients, 78% were admitted to an intensive care unit (ICU), in contrast to 56% of NDM (**Figure 1F**). Additionally, patients with PDM had longer ICU stays than NDM (10 days, IQR 1-17 vs. 2 days, IQR 1-4, respectively, *p* = 0.024) (**Figure 1G**). Regarding mechanical ventilation (MV), 64% of the PDM group required invasive respiratory support, compared to 33% of NDM patients (**Figure 1H**). PDM patients also developed more lung dysfunction based on ratios of oxygen saturation (SpO_2_) (mean ± SD: 243.3 ± 143.7 vs 363.8 ± 130.4, *p* = 0.009) and arterial oxygen partial pressure (PaO_2_) (185.0 ± 105.9 vs 320.5 ± 171.1, *p* = 0.043) to fractional inspired oxygen (FiO_2_) (S/F and P/F ratios, respectively) (**Figures 1I, J**). Together, these findings indicate that PDM is associated with higher IL-6 serum levels and increased risk of severe COVID-19 (lung dysfunction, more frequent and prolonged hospitalization and ICU admission). Our analysis of the expression of gateway receptors for SARS-CoV-2 (ACE2, TMPRSS2 and FURIN) in PBMCs revealed no differences between the groups (see **Supplementary Figure 1**).

### IL-6 Serum Levels as a Biomarker of Severe COVID-19 in Patients With Prediabetes

**Figure 2:**
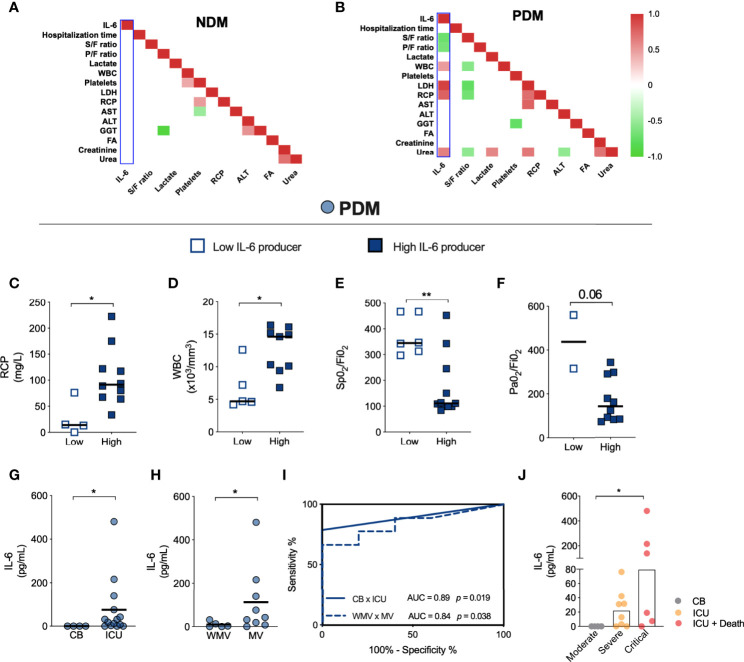
IL-6 production induced by prediabetes dictates the severity of COVID-19. Correlation matrix between clinical parameters of NDM **(A)** and PDM **(B)** patients with COVID-19. Values of **(C)** PCR, **(D)** WBC, **(E)** S/F and **(F)** P/F ratio in PDM patients with low (empty blue box) or high (full blue box) production of IL-6 cytokine. **(G)** IL-6 levels produced by PDM patients hospitalized in CB or ICU. **(H)** IL-6 levels produced by PDM patients who required (MV) or not of ventilation mechanic (WMV). **(I)** Analysis of ROC curve based on IL-6 production in PDM patients in ICU hospitalization (full line) and need for invasive mechanical ventilation (dotted line). **(J)** IL-6 production induced by prediabetes in patients in the moderate, severe and critical form of COVID-19. Correlation positive (red), correlation negative (green). [**(A, B)** = Spearman]; [**(C–H)** = Mann Whitney test]; [**(J)** = Kruskal-Wallis with Dunn’s post-test]. *p < 0.05; **p < 0.01.

We further sought to identify correlations between IL-6 serum levels and different clinical outcomes as well as laboratory parameters. Correlation matrixes constructed for NDM (**Figure 2A**) and PDM (**Figure 2B**) patients revealed no associations regarding IL-6 levels in the NDM group (**Figure 2A** – blue bar). However, positive correlations between IL-6 and WBC (*r* = 0.5*; p* = 0.057), lactate dehydrogenase (LDH) (*r* = 0.9; *p* = 0.001), C-reactive protein (CRP) (*r* = 0.8; *p* = 0.002), and urea (*r* = 0.6; *p* = 0.014) were identified in PDM patients. Additionally, IL-6 was observed to negatively correlate with the S/F and P/F ratio (*r* = -0.7; *p* = 0.002 and -0.6; *p* = 0.023, respectively) in PDM (**Figure 2B**).

Within the PDM patients, a subgroup of 12 patients were observed to produce high levels of IL-6 (termed “high producers”), while 7 had undetectable levels of IL-6 (i.e., “low producers”). We found that the high producers of IL-6 presented increased levels of CRP and WBC (**Figures 2C, D**), while lower S/F and P/F ratios were found in high IL-6 producers (**Figures 2E, F**). Unfortunately, laboratory data was not available for all PDM patients who were low and high IL-6 producers.

We further confirmed that higher levels of IL-6 in PDM patients with COVID-19 was associated with ICU admission (**Figure 2G**) and MV (**Figure 2H**). **Figure 2I** indicates that systemic levels of IL-6 >1.4 pg/mL increase the risk of severe COVID-19 in PDM patients with respect to the outcome of ICU admission (AUC = 0.89; sensitivity of 78.5% and specificity of 100%; *p* = 0.019), while >15.9 pg/mL increased the risk of need for MV (AUC = 0.84; sensitivity of 77.8% and specificity of 80%, LR 3.9; *p* = 0.038) (**Figure 2I**). Finally, high serum levels of IL-6 were also observed in some PDM patients who died following ICU admission (**Figure 2J**). These findings suggest that serum levels of IL-6 are associated with COVID-19 severity in PDM individuals (see **Supplementary Table 2**).

### Prediabetes Does not Worsen Complications in Long-Term COVID-19

**Figure 3:**
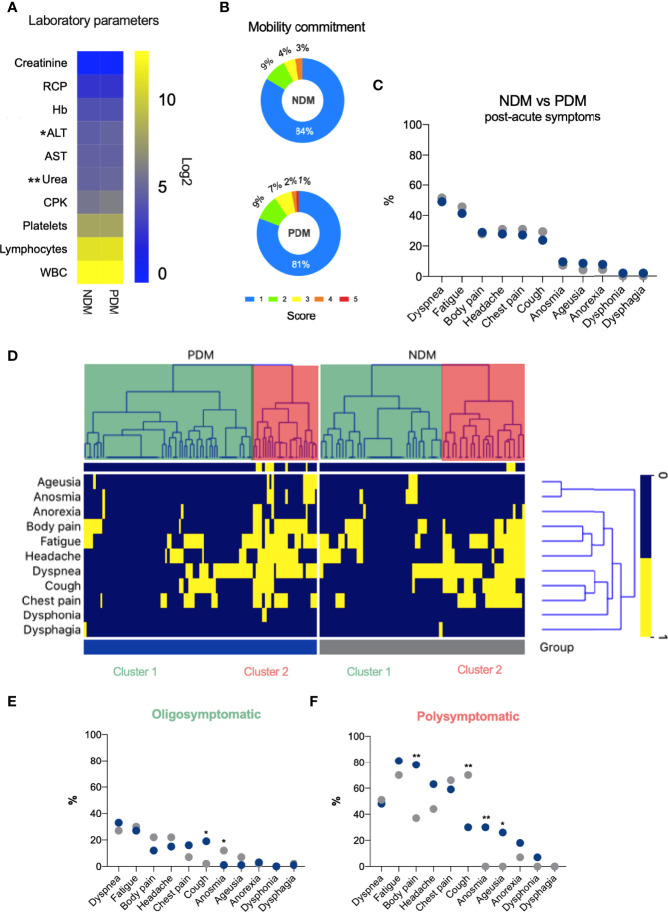
Prediabetes does not alter the symptoms of long-term COVID-19. **(A)** HeatMap shown the median of laboratory parameters values in NDM and PDM patients 3 moths after COVID-19 (blue = low values; yellow = high values). **(B)** Percentage of the degree of mobility impairment in NDM and PDM patients. **(C)** Percentage of symptoms in NDM and PDM patients after COVID-19. **(D)** HeatMap clustered showing symptoms reported by NDM and PDM patients 3 months after COVID-19 (Blue = negative and Yellow = positive for symptoms). **(E)** Percentage of symptoms in NDM and PDM in a population of patients with the highest Percentage of symptoms in NDM and PDM in a population of patients with the highest **(E, F)** percentage of symptoms after COVID-19. (A = Mann Whitney test); (E, F = c² test or Fisher’s exact test). *p < 0.05; **p < 0.01.

We investigated the impact of PDM at 3 months after acute COVID-19 by analyzing laboratory parameters, quality of life and residual symptoms in 130 NDM and 170 PDM patients with post-acute symptoms of COVID-19 (PASC). Patients were matched for age, sex, comorbidities, and disease severity according to ICU admission (**Table 2**). With the exceptions of increased ALT and Urea in PDM patients, no significant differences were noted in the other laboratory parameters analyzed three months following the acute phase of COVID-19 (**Figure 3A**). Despite higher ALT (median of 26 U/L vs 21 U/L, *p* = 0.0185) and Urea (median of 30.0 mg/dL vs 26.5 mg/dL, *p* = 0.0057) levels, values remained within respective reference ranges (See **Supplementary Figure 2**).

**Table 2:** Clinical characteristics between individuals without diabetes (NDM) and with prediabetes (PDM) after 3 moths of the COVID-19 acute phase.

CHARACTERISTICS	Patients, *n*		
	NDM (n = 130)	PDM (n = 173)	*p value*
Male, (%)	56 (43%)	83 (48%)	0.467
Age, mean ± SD	53 ± 12	54 ± 11	0.259
Hb1Ac %, (Min-Max)	5.3 (3.6 - 5.6)	5.9 (5.7 - 6.4)	<0.0001
**Comorbidities n/N (%)**			
Obesity	20/65 (31%)	46/104 (44%)	0.081
Hypertension	46/125 (37%)	67/161 (42%)	0.408
COPD	5/124 (4%)	5/160 (2%)	0.510
**Post-COVID symptoms n/N (%)**			
Dyspnea	35/68 (57%)	51/104 (49%)	0.755
Fatigue	31/68 (45%)	43/104 (41%)	0.582
Headache	21/68 (31%)	29/104 (28%)	0.672
Chest pain	21/68 (31%)	28/103 (27%)	0.600
Cough	20/68 (29%)	25/100 (24%)	0.412
Body pain	19/68 (28%)	30/104 (29%)	0.897
Anosmia	5/68 (7%)	10/104 (10%)	0.607
Ageusia	3/68 (4%)	9/104 (9%)	0.368
Anorexia	3/67 (4%)	8/104 (8%)	0.530
Dysphonia	0/68 (0%)	2/103 (2%)	0.518
Dysphagia	0/68 (0%)	2/103 (2%)	0.518
**Severity n/N (%)**			
Admission to the ICU	32/62 (52%)	57/127 (45%)	0.384

COPD, Chronic Obstructive Pulmonary Disease; ICU, Intensive Care Unit; n, Total number of patients; N, Number of patients with information available.

Based on EuroQol questionnaire results, with scores ranging from 1 (no impairment) to 5 (extremely severe impairment), only 1% of PDM patients reported a score in the mobility dimension (**Figure 3B**). With respect to the other domains, no significant differences were observed (data not shown).

Regarding residual symptoms reported at three months after disease onset, dyspnea (~50%) and fatigue (~43%) were the most frequent, yet no differences among these symptoms were seen between the NDM and PDM patients (**Figure 3C**). For further assessment, an unsupervised analysis was performed to identify hierarchical clustering of symptoms. After removing missing data, 167 patients were analyzed (67 NDM and 100 PDM). Two well-defined clusters were evidenced in both NDM and PDM patients, an oligosymptomatic cluster 1 (green) and a polysymptomatic cluster 2 (red) (**Figure 3D**). The groups were proportionately similar in terms of age, gender, ICU admission and need for invasive mechanical ventilation (IMV) (**Supplementary Table 3**).

In patients with more post-acute COVID-19 symptoms (polysymptomatic), with the exception of cough [30% (PDM) vs 70% (NDM), odds ratio (OR), 0.17, 95% confidence interval (CI) 0.05 to 0.5, *p* = 0.0028], PDM patients more frequently reported body pain (78% vs 37%, OR 5.9, 95% CI 1.82 to 17.81, *p* = 0.0025), anosmia (30% vs 0%, OR infinity, 95% CI 1.9 to infinity, *p* = 0.0100) and ageusia (26% vs 0%, OR infinity, 95% CI 2.543 to infinity, *p* = 0.0043) compared to NDM patients (**Figure 3E**). On the other hand, regarding patients with fewer reported symptoms post-acute COVID-19 (oligosymptomatic), PDM patients reported more coughing (19% vs 2%, OR 9.2, 95% CI 1.60 to 100.5, *p* = 0.0125) and less olfactory dysfunction (1% vs 12%, OR 0.09, 95% CI 0.008 to 0.77, *p* = 0.0116) than NDM (**Figure 3F**).

These findings reveal that despite the presence of post-COVID-19-related symptoms in both groups, differences in the profiles of reported symptoms were evidenced between the polysymptomatic and oligosymptomatic patients. Oligosymptomatic PDM patients present more cough and less anosmia, while more body pain, anosmia, ageusia and less cough were reported by polysymptomatic PDM compared to their respective NDM counterparts. Importantly, no differences in the profile of post-COVID-19-related symptoms were observed in patients admitted to the ICU or those requiring IMV during the acute phase of disease compared to those with milder COVID-19, suggesting that post-acute COVID-19 symptomatology was not dependent on disease severity in the studied patients.

## Discussion

Isolated studies have reported that prediabetes implies an increased risk of severe infection and mortality by COVID-19 (10–12); however, the pathogenic mechanism underlying this risk remains unclear. It was recently demonstrated that 6% to 39.4% of individuals with PDM develop severe COVID-19 (9–11, 13, 14). Although these studies are still scarce, there is accumulating evidence that prediabetes, as well as diabetes, may also culminate in severe COVID-19 (8, 10, 15). The findings reported herein suggest that IL-6 may play a role in the increased risk of severe COVID-19 in PDM individuals. However, it is important to consider that our study was limited in terms of its sample size. PDM patients in the acute phase of infection were older (67 vs 54 years) and more obese (28% vs 0%) than NDM individuals, despite a lack of statistical significance. However, the PDM individuals herein were found to have significantly higher rates of ICU admission and IMV than the NDM patients, which is concordant with age and obesity being known risk factors for severe COVID-19 (16–18).

Previous studies have demonstrated the influence of hyperglycemia on the expression patterns of SARS-CoV-2 gateway receptors, such as ACE2 and TMPRSS2 (3, 4). In this context, differently than COVID-19 patients with diabetes who exhibited increased ACE2 and TMPRSS2 receptor expression in PBMCs, the PDM patients investigated herein had no altered expression patterns for these receptors (3). On the other hand, as with individuals with diabetes, the extent of lung injury (based on the P/F and S/F ratio) was also observed to be significantly greater in patients with prediabetes (3).

Previous studies have reported the impact of hyperglycemia on severe outcomes of COVID-19 in the acute phase, as well the reduced efficiency of tocilizumab therapy, an inhibitor of IL-6 cytokine signaling (19, 20). An exacerbated inflammatory state is known to be one of the main triggers for severe COVID-19 (1). Associations between IL-6 production and COVID-19 severity have been widely reported (1, 3, 21). While the production of this mediator appears to be related to blood glucose levels (22) in the context of COVID-19, no associations with prediabetes have been reported to date (1, 21, 23). Our findings indicate that similarly to diabetic patients, those with prediabetes also induce increased levels of IL-6 during the acute phase of COVID-19 (3, 22). However, while other inflammatory mediators, such as Leukotriene B4, were found to be elevated in patients with diabetes and COVID-19, this was not the case in the PDM patients studied herein (3). As approximately 63% of the PDM patients evaluated produced high levels of IL-6, it is possible that this inflammatory mediator may be a relevant factor in driving patients with prediabetes to develop severe COVID-19 (9–11, 13, 14).

Elevated IL-6 production by individuals with prediabetes was found to correlate with important parameters of severe COVID-19, such as LDH, CRP, and low O_2_ saturation. Koh et al. showed that CRP was a key biomarker associated with diabetes-induced severe COVID-19 (14). In addition, the relationship between IL-6 production and increased CRP has also been reported as a possible trigger for severe COVID-19 (21). LDH has additionally been cited in cases with severe COVID-19, as well as a participation in the activation of inflammasomes, which is related to a worse prognosis of the disease (24, 25). These findings reinforce the notion that enhanced IL-6 production may indeed be associated with a worse prognosis of COVID-19, as the PDM patients evaluated herein produced higher levels of this cytokine non-diabetic COVID-19 patients and were more likely to experience severe outcomes.

Although serious complications have been associated with the acute phase of COVID-19, disease recovery can occur slowly in some cases and may imply residual symptoms, termed “long COVID.” (26, 27) Consistent with our results, Fernández-de-las-Peñas et al. showed that the symptoms reported by patients with diabetes were not different from those without diabetes (28). The present findings indicate that the most frequently reported symptoms in our patients with long COVID-19 were dyspnea, fatigue, headache, cough and body pain, which is consistent with the symptomatology reported in other studies (26, 27, 29).

The persistence of residual symptoms post-COVID-19 requires further study. Importantly, patient sex may be a factor for the reporting of these symptoms regardless of glycemic level (as evidenced by the predominance of females in the polysymptomatic group). Previous reports have argued that sociocultural aspects may be relevant, as women tend to be more concerned with their health (29–31).

In conclusion, despite a relatively limited sample size in the acute phase of SARS-CoV-2 infection, our results indicate that individuals with prediabetes faced an increased risk of developing severe COVID-19, which correlated with high serum levels of IL-6 in these patients. Furthermore, while prediabetes was not shown to significantly exacerbate symptomatology post-acute COVID-19, prediabetic patients present different symptom profiles, depending on their oligosymptomatic and polysymptomatic status, compared to non-diabetic individuals.

## Data Availability Statement

The original contributions presented in the study are included in the article/**Supplementary Material**. Further inquiries can be directed to the corresponding author.

## Ethics Statement

The studies involving human participants were reviewed and approved by The Institutional Board for Ethics in Human Research at the Gonçalo Moniz Institute, Oswaldo Cruz Foundation (CAAE 36199820.6.0000.0040), and Irmã Dulce Social Works (CAAE 33366020.5.0000.0047). The patients/participants provided their written informed consent to participate in this study.

## Author Contributions

IB-S, TC-S, SN, RK, PO, AB, CS, CB, MB-N, VB, and NT contributed to the article’s writing or substantial involvement in its revision before submission. MC, AM, and JC conducted the medical care of the research participants. IB-S, JS, SN, AI and LE processed the biological samples and performed the laboratory essays. IB-S, AM, contributed to the acquisition of the data or the analysis and interpretation of information. IB-S, NT, and VB were involved in the study’s conception, hypotheses delineation, and design. NT is this work’s guarantor. She had full access to all the study’s data and takes responsibility for the integrity of the data and the accuracy of the data analysis. All authors contributed to the article and approved the submitted version.

## Funding

This work was supported by Inova Fiocruz – Oswaldo Cruz Foundation, Coordenação de Aperfeiçoamento de Pessoal de Nível Superior – Brazil (CAPES) under Finance Code 001, Conselho Nacional de Desenvolvimento Científico e Tecnológico – BRAZIL (CNPq) and Fundação de Amparo à Pesquisa do Estado da Bahia - Brazil (FAPESB) project SUS0033/2021. AB, CB, and MB-N are CNPq fellows. NIH grants R01HL124159-01, DK122147-01A1 and AI149207A (to CS).

## Conflict of Interest

The authors declare that the research was conducted in the absence of any commercial or financial relationships that could be construed as a potential conflict of interest.

## Publisher’s Note

All claims expressed in this article are solely those of the authors and do not necessarily represent those of their affiliated organizations, or those of the publisher, the editors and the reviewers. Any product that may be evaluated in this article, or claim that may be made by its manufacturer, is not guaranteed or endorsed by the publisher.

## Supplementary Material

Supplementary Figure 1 Expression of gateway receptors for SARS-CoV-2 is not altered in PBMCs from patients with prediabetes and COVID-19. Gene expression of **(A)** ACE2, **(B)** TMPRRS2 and **(C)** FURIN in peripheral blood mononuclear cells (PBMCs) from patients with COVID-19, without diabetes or with prediabetes.

Supplementary Figure 2 Laboratory parameters after 3 months of the acute phase of COVID-19. Values of **(A)** Urea, **(B)** Alanine aminotransferase, **(C)** Aspartate aminotransferase, **(D)** Creatinofosfoquinase, **(E)** Hemoglobin, **(F)** Cretinine, **(G)** Platelets, **(H)** C-reactive protein, **(I)** White Blood Cells and **(J)** Lymphocytes in NDM and PDM patients 3 moths after COVID-19. Gray region = limit of reference values. Mann Whitney test, **p* < 0.05; ***p* < 0.01.
