# Supplementary figures and images for: Prediabetes Induces More Severe Acute COVID-19 Associated With IL-6 Production Without Worsening Long-Term Symptoms
Source: Front Endocrinol (Lausanne). 2022 Jul 8;13:896378. doi: 10.3389/fendo.2022.896378 (PMC9311489; doi:10.3389/fendo.2022.896378)

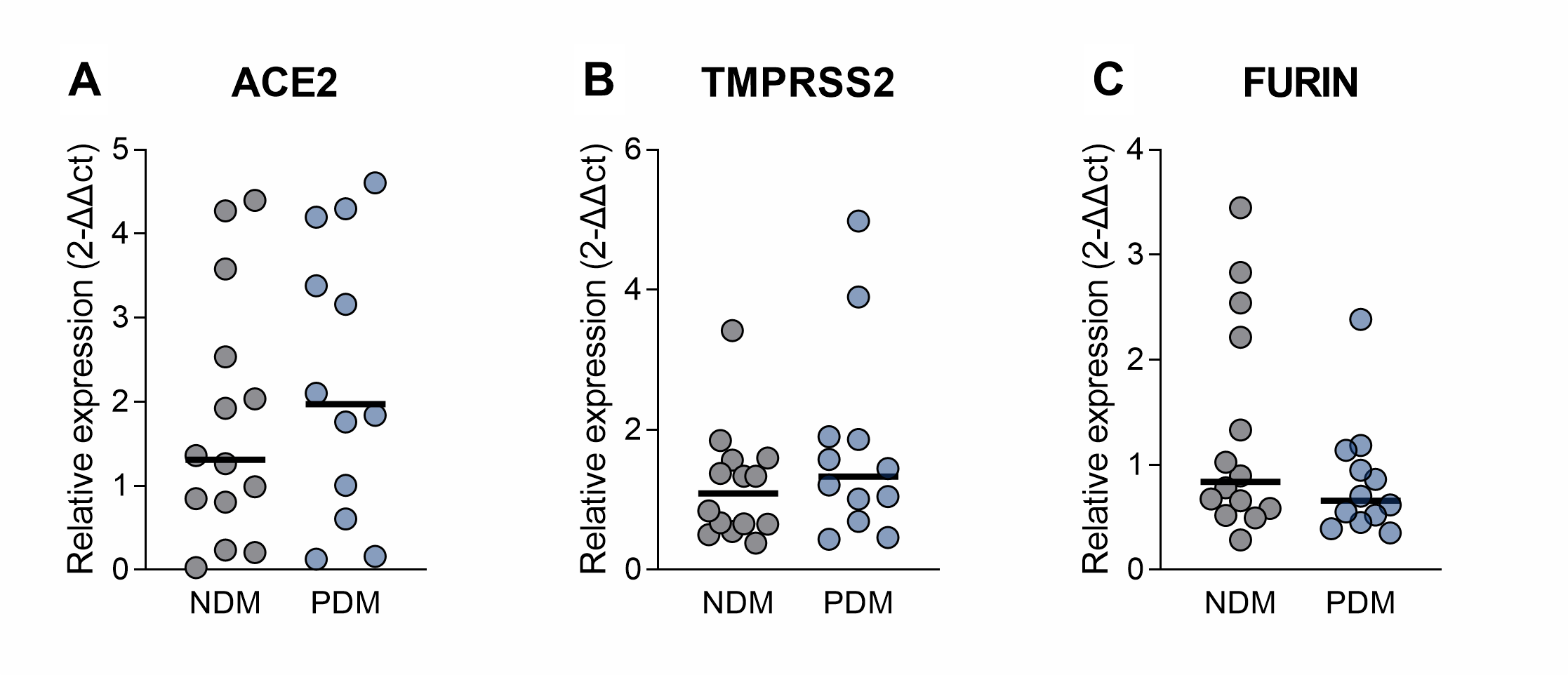

Supplement: Supplementary Figure 1 — Expression of gateway receptors for SARS-CoV-2 is not altered in PBMCs from patients with prediabetes and COVID-19. Gene expression of (A) ACE2, (B) TMPRRS2 and (C) FURIN in peripheral blood mononuclear cells (PBMCs) from patients with COVID-19, without diabetes or with prediabetes. [file Image_1.tif]

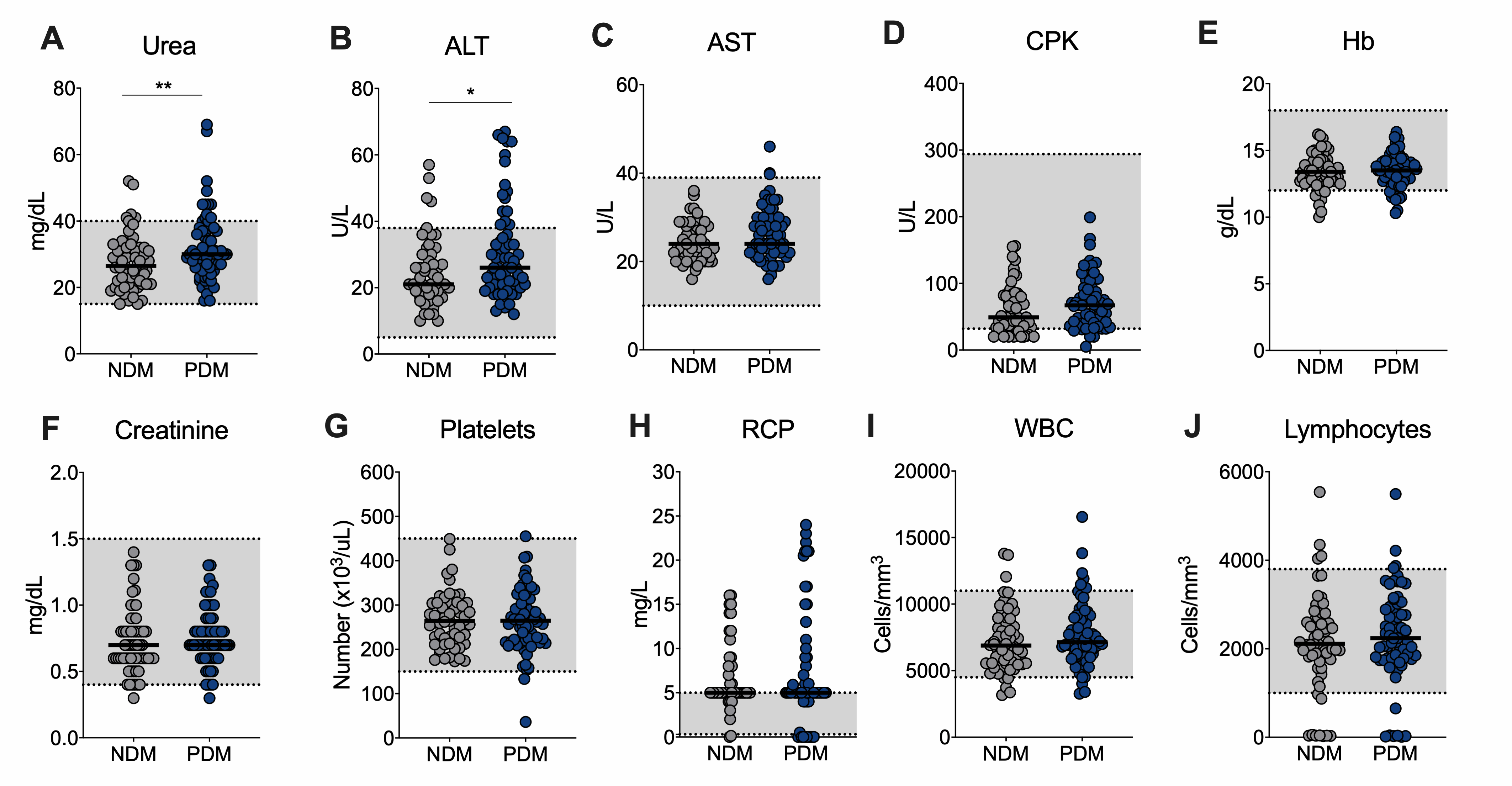

Supplement: Supplementary Figure 2 — Laboratory parameters after 3 months of the acute phase of COVID-19. Values of (A) Urea, (B) Alanine aminotransferase, (C) Aspartate aminotransferase, (D) Creatinofosfoquinase, (E) Hemoglobin, (F) Cretinine, (G) Platelets, (H) C-reactive protein, (I) White Blood Cells and (J) Lymphocytes in NDM and PDM patients 3 moths after COVID-19. Gray region = limit of reference values. Mann Whitney test, *p < 0.05; **p < 0.01. [file Image_2.tiff]
