# Supplementary material for: Prediabetes Induces More Severe Acute COVID-19 Associated With IL-6 Production Without Worsening Long-Term Symptoms
Source: Front Endocrinol (Lausanne). 2022 Jul 8;13:896378. doi: 10.3389/fendo.2022.896378 (PMC9311489; doi:10.3389/fendo.2022.896378)
Supplement: Supplementary file 3 [file Table_1.docx]

**Table S1. Medications used during hospitalization for COVID-19 in patients without diabetes (NDM) and with prediabetes (PDM).**

|  | **Patients, *n*** | | |
| --- | --- | --- | --- |
| **CHARACTERISTICS** | **NDM (n = 23)** | **PDM (n = 19)** | *p value* |
| Systemic corticosteroid n, N (%) | 13/22 (59%) | 13/16 (81%) | 0.178 |
| Anticoagulant n, N (%) | 16/20 (80%) | 13/14 (93%) | 0.378 |
| Dobutamine n, N (%) | 0/22 (0%) | 3/17 (18%) | 0.074 |
| Noradrenaline n, N (%) | 5/22 (23%) | 9/17 (53%) | 0.091 |
| Antiviral n, N (%) | 5/21 (24%) | 7/15 (47%) | 0.175 |

n = Positive numbers; N = Number available
