# Supplementary material for: Prediabetes Induces More Severe Acute COVID-19 Associated With IL-6 Production Without Worsening Long-Term Symptoms
Source: Front Endocrinol (Lausanne). 2022 Jul 8;13:896378. doi: 10.3389/fendo.2022.896378 (PMC9311489; doi:10.3389/fendo.2022.896378)
Supplement: Supplementary file 4 [file Table_2.docx]

**Table S2. Multivariate logistic regression analysis for severe COVID-19 outcome.**

| **Characteristic** | **OR^1^** | **95% CI^1^** | **p-value** |
| --- | --- | --- | --- |
| **Sex** |  |  |  |
| Female | — | — |  |
| Male | 0.86 | 0.16, 4.35 | 0.9 |
| **Age (years)** | 0.98 | 0.94, 1.02 | 0.4 |
| **Condition** |  |  |  |
| NDM | — | — |  |
| PDM | 2.27 | 0.38, 15.3 | 0.4 |
| **IL-6 Producer (yes)** | 19.1 | 2.71, 401 | 0.012 |
