# Supplementary material for: Prediabetes Induces More Severe Acute COVID-19 Associated With IL-6 Production Without Worsening Long-Term Symptoms
Source: Front Endocrinol (Lausanne). 2022 Jul 8;13:896378. doi: 10.3389/fendo.2022.896378 (PMC9311489; doi:10.3389/fendo.2022.896378)
Supplement: Supplementary file 5 [file Table_3.docx]

**Table S3. Sociodemographic characteristics of oligosymptomatic and polysymptomatic patients with symptoms after 3 months of COVID-19 acute phase.**

|  | **Oligosymptomatic** | | | **Polysymptomatic** | | |
| --- | --- | --- | --- | --- | --- | --- |
| **CHARACTERISTICS** | **NDM (n = 40)** | **PDM (n = 73)** | *p value* | **NDM (n = 27)** | **PDM (n = 27)** | *p value* |
| Male, n/N (%) | 25/40 (62%) | 41/73 (56%) | 0.513 | 7/27 (26%) | 7/27 (26%) | >0.999 |
| Age, mean ± SD | 53 ± 10 | 56 ± 12 | 0.222 | 53 ± 10 | 53 ± 10 | 0.813 |
| Admission to ICU n, N (%) | 14/22 (64%) | 27/61 (44%) | 0.119 | 6/13 (46%) | 7/18 (37%) | 0.598 |
| IMV required n, N (%) | 5/14 (36%) | 6/27 (22%) | 0.462 | 3/6 (50%) | 2/7 (28%) | 0.592 |

ICU = Intensive Care Unit; IMV = Invasive Mechanical Ventilation; n = Total number of patients; N = Number of patients with information available.
